# Poor semen quality is associated with impaired antioxidant response and acute phase proteins and is likely mediated by high cortisol levels in *Brucella*-seropositive dromedary camel bulls

**DOI:** 10.1038/s41598-024-74018-y

**Published:** 2024-11-13

**Authors:** Ahmed Saad Ahmed Hassaneen, Anis Anis, Safaa Y. Nour, Rasha Salah Mohamed, Islam M. Wassif, Adel M. El-kattan, Hosny Ahmed Abdelgawad, Ragab H. Mohamed

**Affiliations:** 1 Department of Theriogenology, Obstetrics, and Artificial Insemination, Faculty of Veterinary Medicine, South Valley University, Qena, 83523 Egypt; 2 Department of Pathology, Faculty of Veterinary Medicine, University of Sadat City, Sadat City, Monufia 32897 Egypt; 3 Department of Animal Medicine, Faculty of Veterinary Medicine, Aswan University, Aswan, 81511 Egypt; 4 Department of Animal and Poultry Health, Desert Research Center, Cairo, Egypt; 5 Department of Microbiology and Immunology, Faculty of Veterinary Medicine, Aswan University, Aswan, 81511 Egypt; 6 Department of Theriogenology, Faculty of Veterinary Medicine, Aswan University, Aswan, 81511 Egypt

**Keywords:** Antioxidants, Brucellosis, Camels’ infectious disorders, Dromedary bulls infertility, Microbiology, Zoology

## Abstract

Brucellosis in dromedary camel bulls leads to either temporary or permanent loss of fertility. Camel brucellosis is associated with both orchitis and epididymitis. However, the clinical signs of camel brucellosis are not clear as those in cattle. Therefore, this study aimed to diagnose camel brucellosis based on a serological screening using Rose Bengal plate test (RBPT) followed by competitive ELISA. To understand the impact of brucellosis on camel bull fertility, this study aimed to examine the semen characteristics, evaluate the testicular histopathology, examine hormonal profile, antioxidants and acute phase proteins (APP). A total of 150 mature bulls were used in this study. Blood samples were collected for serological, hormonal, and biochemical analysis. This study revealed that 6.6% and 7.3% of the examined bulls were *Brucella*-seropositive using RBPT and competitive ELISA, respectively. The *Brucella*-seropositive dromedary bulls showed poor semen quality, pathological changes orchitis, and lower testosterone. Moreover, our findings showed a higher cortisol level, and significant impairments in the measured APP and antioxidants in *Brucella*-seropositive bulls. In conclusion, the *Brucella*-seropositive dromedary bulls showed lower fertility due to poor semen quality and lower testosterone levels. Such lower fertility is likely mediated by high cortisol levels, and impaired APP and antioxidants’ defense response.

**Supplementary Information:**

The online version contains supplementary material available at 10.1038/s41598-024-74018-y.

## Introduction

Dromedary Arabian camels are distributed in many tropical and sub-tropical countries in Africa and Asia. They are raised for meat production, milk production, transportation, and racing^1–3^. Dromedary camels are short-day seasonal breeder animals that have a relatively short breeding season that extends from December to February^4–6^. In addition to such a relatively short breeding season, dromedary camels are characterized by poor fertility^3^.

Camels are not resistant to many infectious diseases affecting other livestock with a high susceptibility to different bacterial pathogens including *Brucella* species^7,8^. Infectious diseases that are affecting reproductive system led to either temporary or permanent loss of fertility^9,10^. Camel infertility caused by brucellosis is associated with orchitis, epididymitis and abnormal semen picture^10^, in addition to locomotor disorders due to arthritis and hygroma^10,11^. A global meta-epidemiological study on camel brucellosis reported that the overall prevalence of camel brucellosis worldwide was about 9.2%^12^.

Diagnosis of camel brucellosis has some difficulties because the clinical signs are not clear as those in cattle^12–15^. Therefore, the need for accurate diagnostic method(s) is necessary. Different serological tests were reported to be used for diagnosis of camel Brucellosis. A combination of two serological tests of high sensitivity and specificity confirms accuracy of diagnosis^16^. In eradication efforts or for international trade testing, the Rose Bengal test/complement fixation test (RBT/CFT) combination and the indirect (iELISA) or competitive (cELISA) enzyme-linked immunosorbent tests are frequently performed^17^. Rose Bengal test and cELISA were suitable for diagnosis of Brucellosis in camel sera^18^. It was reported that a combination of direct methods and indirect methods are effective to detect positive all cases of camel brucellosis^19^.

In response to bacterial infections, production of acute phase proteins (APP) is important as a non-specific early defense system prior to proper immunological reactions. Acute phase proteins activate the immune system and enhance phagocytosis^20–22^. The APP including fibrinogen and haptoglobin are not only used as non-specific diagnostic markers but also as prognostic markers of infections^20,23–25^. Reactive oxygen species (ROS), also known as oxygen free radicals, are natural by-products of cellular response to bacterial infections. However, an access of free radicals is causing oxidative damage^26,27^. Appropriate antioxidants’ defense response is critical to overcome such oxidative damage. The imbalance between the cellular antioxidant defense systems and the production of ROS is known as oxidative stress^28^. Oxidative stress is one of the early events in disease development^29^. Non-enzymatic antioxidants such as ascorbic acid and enzymatic antioxidants such as superoxide dismutase (SOD) are very important to defend against oxidative stress. It should be noted that, the enzymatic antioxidants activity is increased in response to vaccination with *Brucella*^30^. The present study aimed to serologically screen dromedary bulls for brucellosis, and to evaluate the brucellosis-associated testicular pathological changes, hormonal changes, and antioxidant status. To achieve this aim, the present study evaluated semen characteristics, hormonal profiles, APP, and antioxidants (non-enzymatic; ascorbic acid, and enzymatic; SOD). In addition, the present study aimed to study the possible correlations among these evaluated parameters in the examined bulls to provide better understanding about brucellosis in dromedary bulls.

## Results

### Serological findings for brucellosis

Out of 150 serum samples of the examined bulls screened for *Brucella* antibodies, 10 (6.6%) and 11 (7.3%) were positive by RBPT and cELISA, respectively. Four out of the *Brucella*-seropositive-bulls showed orchitis and lameness.

### Semen picture in *Brucella*-seropositive and seronegative dromedary bulls

**Fig. 1:**
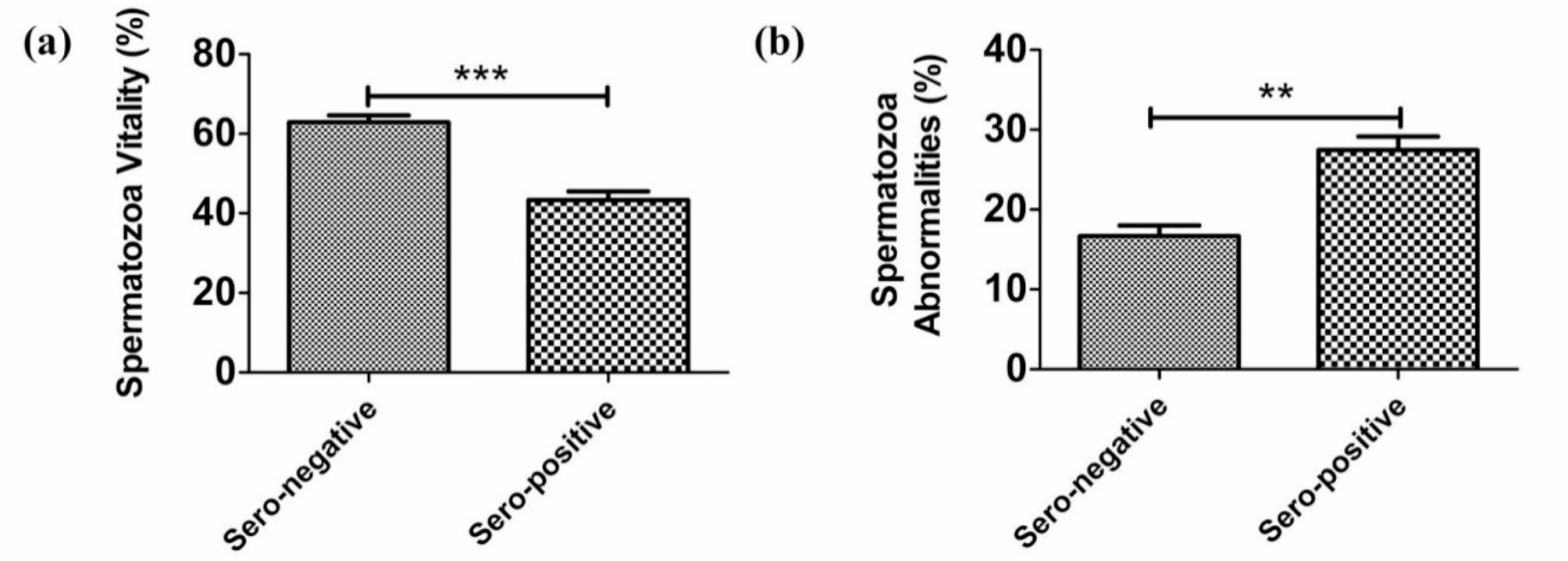
Semen analysis of epididymal semen in both *Brucella*-seropositive and seronegative dromedary bulls. (**a**) Spermatozoa vitality (%). (**b**) Spermatozoa abnormalities (%). (**) means significant at < 0.01, (***) means significant at < 0.001.

The vitality of spermatozoa (%; mean ± SEM) in *Brucella*-seropositive dromedary bulls was lower (43.35 ± 2.16%, *P* < 0.001) than that in the *Brucella*-seronegative bulls (62.92 ± 4.59%) (Fig. 1a). In addition, the spermatozoa abnormalities (%; mean ± SEM) in the *Brucella*-seropositive bulls were higher (27.44 ± 1.71%, *P* < 0.01) than those in the *Brucella*-seronegative bulls (16.67 ± 1.35%) (Fig. 1b).

### Serum testosterone and cortisol in *Brucella*-seropositive and seronegative dromedary bulls

**Fig. 2:**
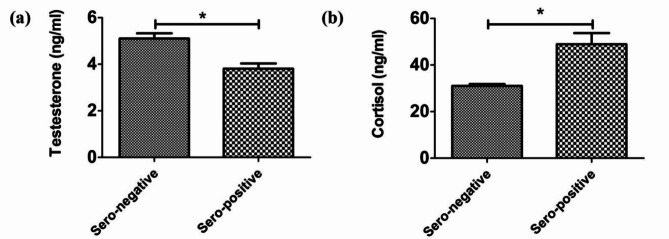
Serum levels of the measured steroid hormones in both *Brucella*-seropositive and *Brucella*-seronegative dromedary bulls. (**a**) Testosterone hormone concentration (ng/ml), (**b**) cortisol hormone concentration (ng/ml). (*) means significant at < 0.05.

The serum testosterone concentration (ng/ml; mean ± SEM) in the *Brucella*-seropositive dromedary bulls was lower (3.81 ± 0.22 ng/ml, *P* < 0.05) than that in the *Brucella*-seronegative bulls (5.10 ± 0.23 ng/ml) (Fig. 2a). While the serum cortisol concentration (ng/ml; mean ± SEM) in the *Brucella*-seropositive bulls was higher (48.93 ± 4.81 ng/ml, *P* < 0.05) than that in the *Brucella*-seronegative bulls (31.00 ± 0.81 ng/ml) (Fig. 2b).

### Concentrations of serum antioxidants in *Brucella*-seropositive and seronegative dromedary bulls

The serum concentration of SOD (U/ml; mean ± SEM) was lower (255.30 ± 1.98 U/ml, *P* < 0.001) in the *Brucella*-seropositive dromedary bulls than that in the *Brucella*-seronegative bulls (273.10 ± 1.72 U/ml) (Table 1). However, the serum concentration of ascorbic acid (ng/dl; mean ± SEM) in the *Brucella*-seropositive bulls was higher (137.60 ± 2.10 ng/dl, *P* < 0.05) than that in the *Brucella*-seronegative bulls (122.60 ± 1.38 ng/dl) (Table 1).

### Concentrations of serum APP in *Brucella*-seropositive and seronegative dromedary bulls

The serum concentration of fibrinogen (mg/dl; mean ± SEM) was higher (337.60 ± 4.12 mg/dl, *P* < 0.01) in the *Brucella*-seropositive dromedary bulls than that in the *Brucella*-seronegative bulls (311.50 ± 1.64 mg/dl) (Table 1). However, the serum concentration of haptoglobin (mg/dl; mean ± SEM) was lower (8.80 ± 0.23 mg/dl, *P* < 0.05) in the *Brucella*-seropositive bulls than that in the *Brucella*-seronegative bulls (10.40 ± 0.21 mg/dl) (Table 1).

**Table 1:** Serum levels of antioxidants and acute phase proteins in both *Brucella*-seronegative and *Brucella*-seropositive dromedary bulls.

	Antioxidants		Acute phase proteins	
	Ascorbic acid (ng/dl)	Superoxide dismutase (SOD; U/ml)	Fibrinogen (mg/dl)	Haptoglobin (mg/dl)
Seronegative-dromedary bulls	122.60 ± 1.38	273.10 ± 1.72***	311.50 ± 1.64	10.40 ± 0.21*
Seropositive-dromedary bulls	137.60 ± 2.10*	255.30 ± 1.98	337.60 ± 4.12**	8.80 ± 0.23

(*) means significant at < 0.05, (**) means significant at < 0.01, (***) means significant at < 0.001.

### Correlations between semen evaluation parameters, steroid hormones, APP, and antioxidants biomarkers in all the examined *Brucella*-seronegative and *Brucella*-seropositive dromedary bulls

Significant correlations were reported between epididymal semen quality parameters and steroid hormones, APP, and antioxidant biomarkers (Table 2). Positive correlations were demonstrated between spermatozoa vitality and testosterone, SOD, and haptoglobin. Negative correlations were reported between spermatozoa vitality and spermatozoa abnormalities, cortisol, fibrinogen, and ascorbic acid. Negative correlations were reported between spermatozoa abnormalities and both haptoglobin and SOD. Positive correlations were demonstrated between spermatozoa abnormalities and cortisol, fibrinogen, and ascorbic acid (Table 2).

Significant correlations were reported between antioxidants and APP biomarkers (Table 2). Positive correlations were demonstrated between ascorbic acid and both cortisol, fibrinogen. Negative correlations were reported between ascorbic acid and both haptoglobin and SOD. Negative correlations were reported between SOD and both haptoglobin and fibrinogen. And a negative correlation was also demonstrated between haptoglobin and fibrinogen (Table 2).

**Table 2:**
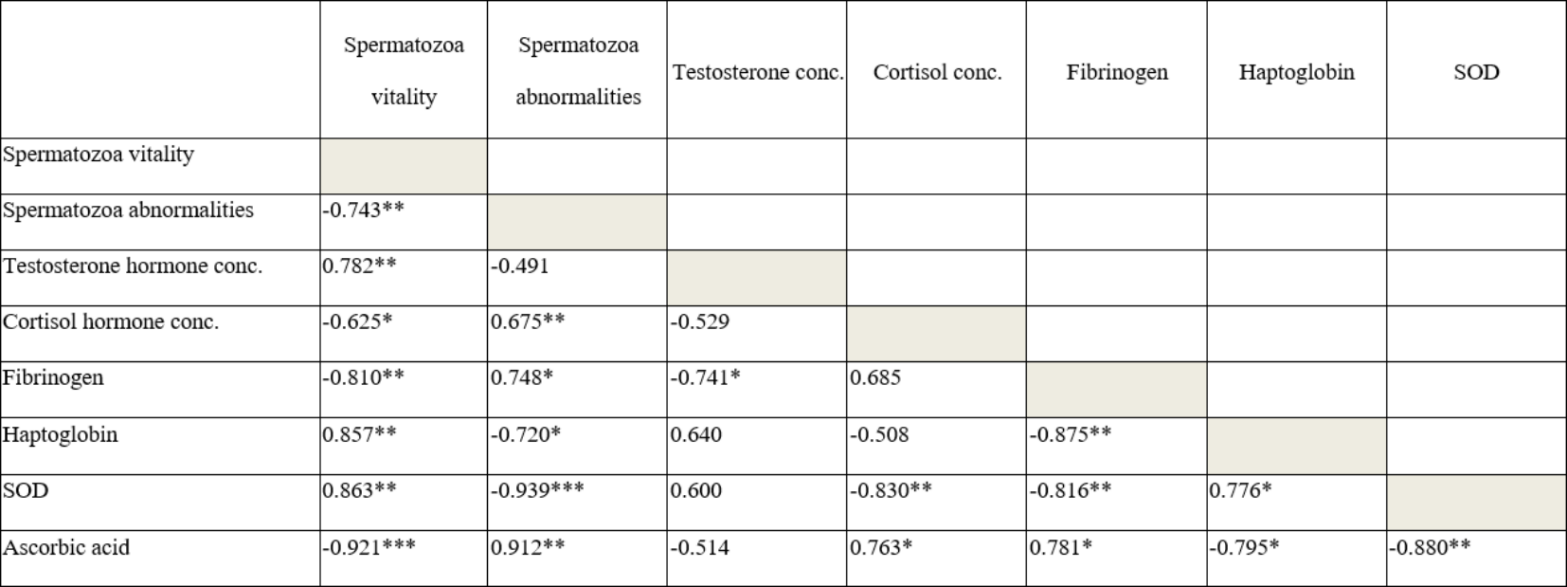
Pearson correlation coefficients between semen parameters, hormones, acute phase proteins, and antioxidants in all dromedary bulls.

Some serum samples could not be evaluated for the antioxidants and acute phase proteins parameters. the values of these samples were kept blank during the statistical analysis of the correlation coefficients.

### Histopathological changes in the testes of *Brucella*-seropositive dromedary bulls

**Fig. 3:**
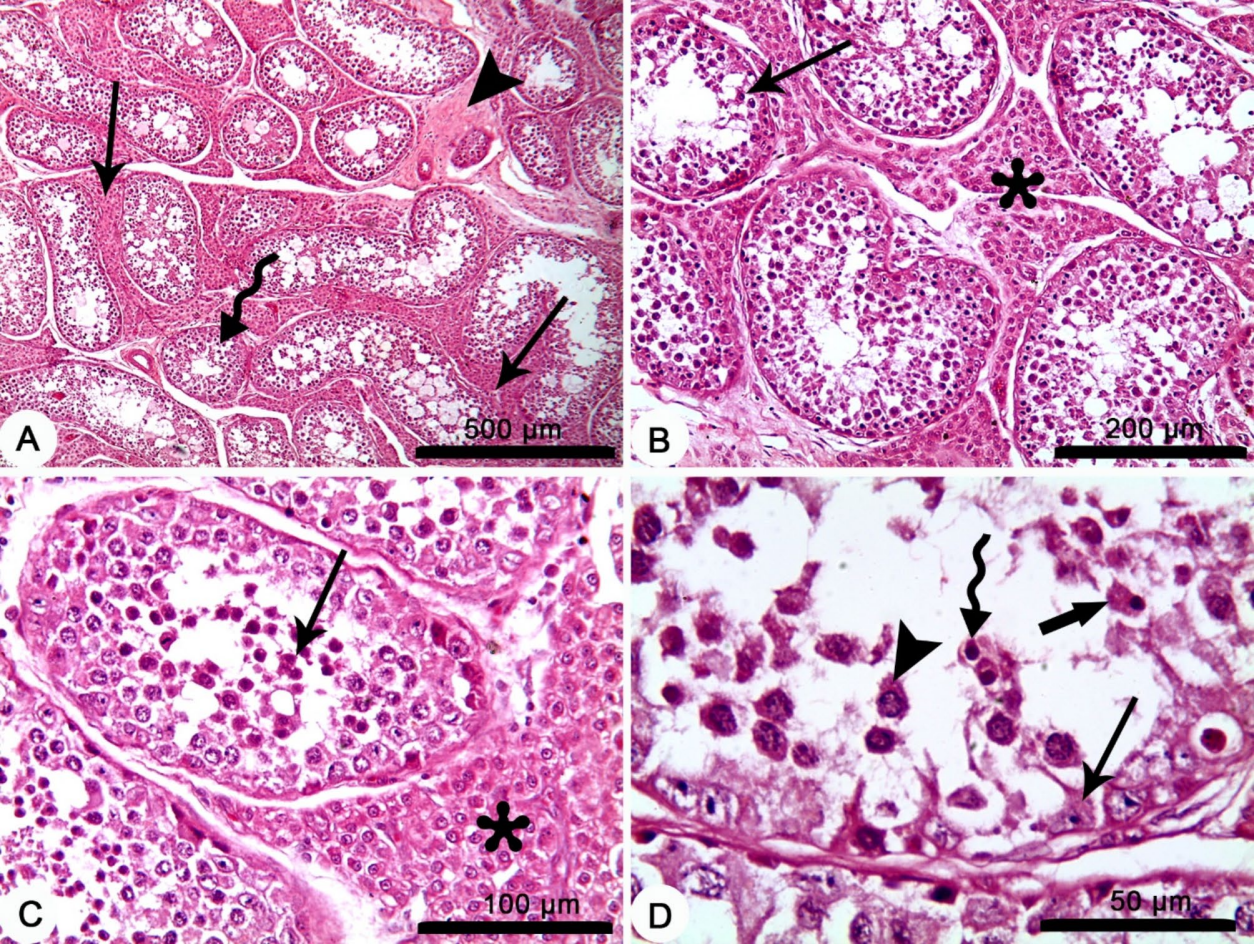
Representative images for the histopathological changes in the testes of a *Brucella*-seropositive dromedary bull showing subacute orchitis. (**A**) Widespread infiltration of inflammatory cells in the interstitial tissues (arrows), interstitial edema (arrowhead) and decreased size of seminiferous tubules (bended arrow). (**B**) Heavy infiltration of the interstitial tissue with inflammatory cells (asterisk) and degeneration of the epithelium lining of seminiferous tubules (arrow). (**C**) Heavy infiltration with inflammatory cells in the interstitial tissue with (asterisk) and in the lumen of seminiferous tubules (arrow). (**D**) Degeneration of germ cell layer (thin arrow), infiltration of macrophages (thick arrow), lymphocyte (bended arrow) and Neutrophils (arrowhead) in the lumen of seminiferous tubules. H&E stain.

**Fig. 4:**
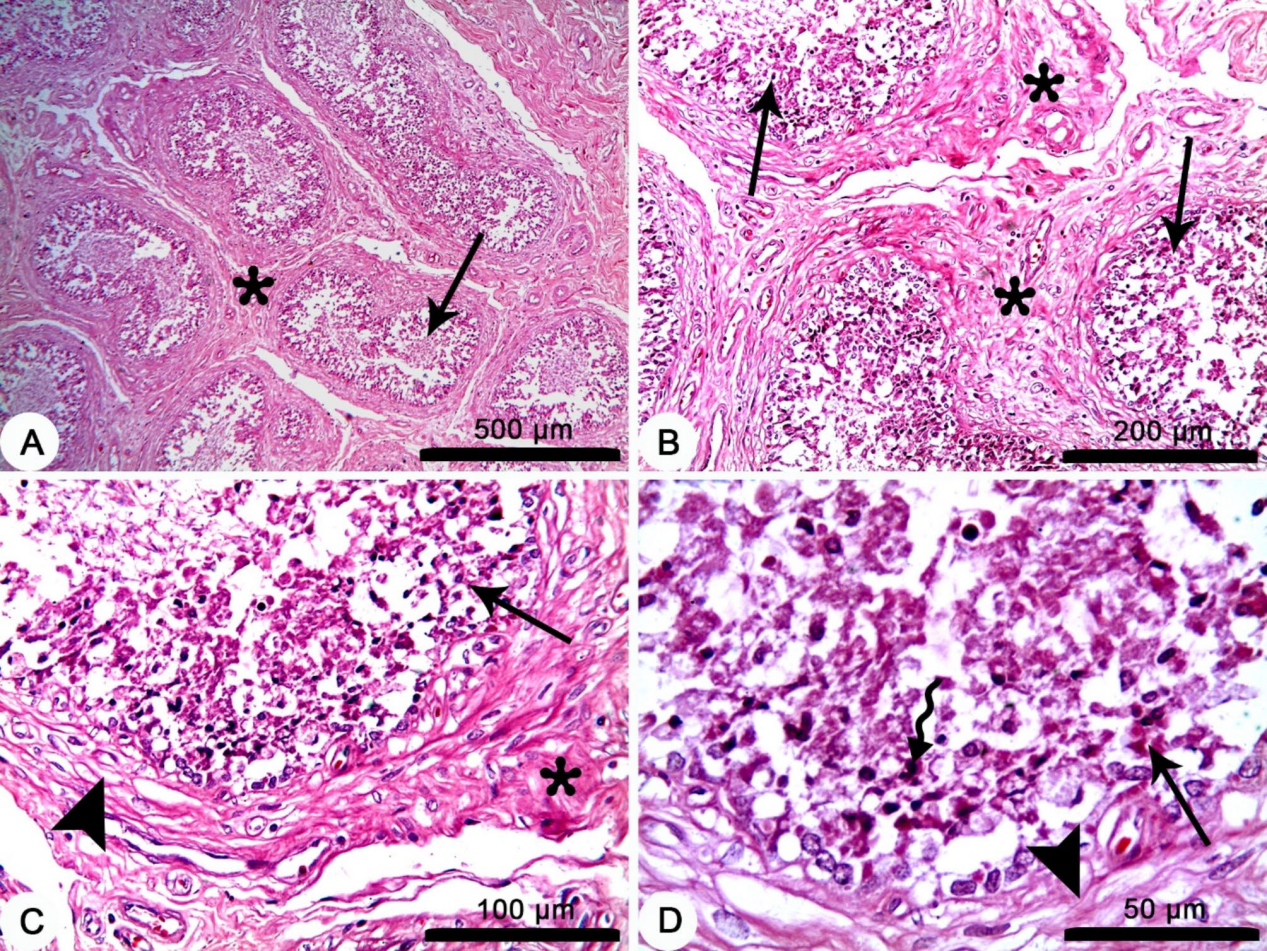
Representative images for the histopathological changes in the testes of a *Brucella*-seropositive dromedary bull showing chronic fibrosed orchitis. (**A**) Widespread interstitial fibrosis (asterisk) between atrophied seminiferous tubules (arrow). (**B**) Widespread interstitial fibrosis (asterisks) and atrophied seminiferous tubules with degeneration and necrosis of the lining epithelium (arrows). (**C**) Intensive fibrosis in the interstitial tissue (asterisks), peritubular fibrosis (arrowhead) and necrosis of tubular lining epithelium (arrows). (**D**) Peritubular fibrosis (arrowhead), necrosis of tubular lining epithelium (arrows) and lymphoid cells’ infiltration in the tubular lumen (bended arrow). H&E stain.

The descriptive histopathological examination of the testes in the *Brucella*-seropositive dromedary bulls showed variable pathological changes of subacute and chronic orchitis. Affected testes of *Brucella*-seropositive bulls (*n* = 2) showed pathological changes of subacute orchitis with widespread and heavy infiltrations of inflammatory cells in the interstitial tissues, interstitial edema, decreased size and degeneration of the epithelium lining of seminiferous tubules with degeneration of germ cell layer (Fig. 3). While affected testes of another *Brucella*-seropositive bulls (*n* = 2) showed pathological changes of chronic orchitis with widespread interstitial fibrosis between atrophied seminiferous tubules, with degeneration and necrosis of the lining epithelium, and lymphoid cells’ infiltrations in the tubular lumen (Fig. 4).

## Discussion

Due to the increasing demands for animal proteins, maintenance of both camels’ productive and reproductive performance is important. More research groups are focusing on different aspects related to reproductive performance and infertility problems in camels^8,27,31,32^. However, information on the infectious infertility disorders such as brucellosis is still not fully covered in camels. Therefore, the findings of the current study successfully determined the semen characteristics, APP, antioxidants, and hormonal profile in *Brucella*-seropositive bulls and the associated pathological changes in the testes. Moreover, this study found a strong relation between the semen quality parameters and the measured hormonal, APP and antioxidants parameters.

The serological screening in the present study reported a prevalence of camel brucellosis about 6.6% and 7.3% by using RBPT and cELISA, respectively. Similar seroprevalence of 6.5% was recently reported in dromedary bulls in Qatar^33^. Lower rates of prevalence (4.4% and from 3.73 to 4.17%) were previously reported by in Abu Dhabi and Egypt, respectively^34,35^. However, a higher seroprevalence (12.9%) by RBPT was recorded in Egypt^36^ in a prevalence study conducted on camel population in Red Sea governorate during the period from 2014 to 2015.

The evaluated semen characteristics (spermatozoa viability and spermatozoa abnormalities) were negatively affected in the *Brucella*-seropositive bulls, that the *Brucella*-seropositive bulls showed lower spermatozoa viability (*P* < 0.001) and higher spermatozoa abnormalities (*P* < 0.01) compared with the *Brucella*-seronegative bulls. When compared to the *Brucella*-seronegative bulls, the serum testosterone levels (ng/ml) were significantly lower (*P*< 0.05). It is well known that testosterone is responsible for the reproductive performance in male animals including dromedary bulls, such reproductive performance includes libido, rutting behavior, and normal function of accessory genital glands^31,37–39^. The lower serum testosterone levels reported in the present study come in line with the poor semen quality parameters that the present study found in the *Brucella*-seropositive bulls.

Significantly higher serum cortisol levels (ng/ml) in the *Brucella*-seropositive bulls reported in the current study is likely related with a negatively affected sexual behavior in male camels^40^ and poor semen quality reported in the present study. Both findings in the previous and the present study support that higher cortisol levels negatively affect reproductive performance of camel bulls.

On the other hand, *Brucella*-seropositive bulls showed variable antioxidant response when compared to that of *Brucella*-seronegative bulls with lower (*P* < 0.001) level of SOD (U/ml) and higher (*P* < 0.05) level of ascorbic acid (ng/dl). Similar findings were recently reported in *Brucella*-seropositive ewes where the naturally *Brucella*-infected ewe showed lower levels of SOD and increased levels of ascorbic acid^41^. The increased levels of ascorbic acid in *Brucella*-seropositive camels would be due to increase the hepatic synthesis of the ascorbic acid as an antioxidant protective response against the oxidative stress^30,42^. While, the lower levels of SOD would be likely associated with the inability of cytokines activation^43^. It should be noted that, in a previous clinical study, *Brucella*-seropositive patients also showed lower levels of SOD^44^.

The APP levels were significantly different in *Brucella*-seropositive bulls when compared to those of *Brucella*-seronegative bulls with lower (*P* < 0.05) level of haptoglobin (mg/dl) and higher (*P*< 0.001) level of fibrinogen (mg/dl). The lower levels of haptoglobin would be due to depletion of haptoglobin associated with chronic/prolonged infection^12,45^. Higher fibrinogen levels are likely due to the increase of fibrinogen synthesis in response to tissue damage caused by brucellosis^46^. A previous study reported no significant difference in fibrinogen levels between both *Brucella*-seropositive and *Brucella*-seronegative male camels^12^. However, it is well known that increased fibrinogen levels are generally associated with different forms of inflammation^12,41,47^.

The testicular pathological changes of decreased size or even atrophied seminiferous tubules with degeneration and necrosis of the lining epithelium found in both the subacute and chronic orchitis support the present finding of poor semen quality with lower spermatozoa vitality and higher spermatozoa abnormalities. The testicular pathological changes reported in this study is mediated by impaired antioxidant response and increase the ROS the cause oxidative damage. Moreover, spermatozoa are highly susceptible to damage induced by ROS. This is likely due to the high content of polyunsaturated fatty acids within the plasma membranes of spermatozoa and low levels of enzymatic antioxidants^48–50^. On the other hand, it should be noted that signs of chronic orchitis including atrophied seminiferous tubules have been detected in *Brucella*-seronegative camels^51^. This is likely because of low antibody levels during chronic infections with brucellosis^52^. There is another possibility, that the *Brucella*-seronegative camels were infected by other orchitis-causing infections or traumatic injury.

Moreover, the intensive fibrosis in the interstitial tissue would be the reason of lower testosterone levels in the *Brucella*-seropositive bulls as the interstitial Leydig cells are the main source of testosterone hormone^50^.

Significant correlations were reported between the evaluated semen parameters and the other measured serum parameters; steroid hormones, antioxidants and APP. The evaluation of biochemical and hormonal parameters serum would be a reflection to the levels of these parameters in epididymal seminal fluid, in camels^31^. Positive correlations were demonstrated between the spermatozoa vitality and testosterone hormone, haptoglobin, and SOD. These positive correlations are supported by the present findings of lower spermatozoa vitality, testosterone concentration, haptoglobin and SOD in *Brucella*-seropositive camel bulls. Negative correlations were reported between spermatozoa vitality and spermatozoa abnormalities, cortisol, fibrinogen, and ascorbic acid. On the other hand, positive correlations were reported between spermatozoa abnormalities and cortisol, fibrinogen, and ascorbic acid. While negative correlations were demonstrated between spermatozoa abnormalities and haptoglobin, and SOD. It was suggested that the low levels of the reproductive hormone reported in infertile male camels would be associated with spermatozoa abnormalities due to oxidative stress resulted from the infection in dromedary bulls^53^. The correlations reported in our present study strongly support the other finding of higher cortisol levels and poor semen quality in *Brucella*-seropositive camel bulls. Moreover, the increased levels of fibrinogen and ascorbic acids were found in *Brucella*-seropositive camels in order to combat oxidative stress to improve the correlated poor semen quality. This notion is supported by the negative correlation between fibrinogen and SOD, previously reported^54^.

In conclusion, the *Brucella*-seropositive dromedary bulls that suffered from either subacute or chronic orchitis showed lower fertility due to both poor semen quality and lower testosterone levels. Such lower fertility is likely mediated by high cortisol levels, and the changes in APP and antioxidant biomarkers’ concentrations. The present study reported significant correlations between the semen picture and the other measured parameters, steroid hormones, antioxidant biomarkers and APP.

## Methods

The current study was performed during the period from December 2020 to September 2022.

### Ethics approval

This study was performed in line with the principles of the Declaration of Helsinki and complied with the ARRIVE guidelines and all methods were conducted following relevant guidelines and regulations. The present study was approved by the Ethical Research Committee of the Faculty of Veterinary Medicine, South Valley University, Qena, Egypt (final approval number 61/18.09.2022, approved September 18, 2022).

### Animals

This study used a total of 150 mature dromedary camel bulls aged 5–12 years-old (Supplementary Figure S1) during the period from 2020 to 2021. All bulls were admitted for slaughtering at the local abattoirs, Aswan governorate, Egypt. The used animals were subjected to clinical examination under appropriate precaution measures.

### Blood sampling

Blood samples were collected from the jugular vein using plain vacutainer tubes and the sera were separated by centrifugation at 3000 rpm for 20 min. Separated serum samples were divided into aliquots and stored at -20 °C until further serological, hormonal, and biochemical analysis.

### Serological allocation of dromedary bulls

After serological screening, the bulls were divided into two groups: *Brucella*-seropositive and *Brucella*-seronegative bulls. The serological allocation was maintained by Rose Bengal plate test^55^ as primary screening and confirmed by competitive ELISA (COMPELISA 400^®^, APHA, New Haw, Addlestone, U.K.).

### Semen collection and analysis

Semen samples were collected from the epididymis of the examined bulls as previously described. In brief, the epididymis was dissected and the epididymal tail was incised longitudinally and rinsed 3–4 times.

using Brackett and Oliphant medium in Petri dishes of 60-mm size (Liverpool, Australia, Bacto Lab.) and placed on a warm stage (37 °C) to obtain a sperm-rich fluid^56,57^. The sperm rich fluid is examined for the vitality of spermatozoa using 0.5% eosin and 10% nigrosine stains^58^, and for the spermatozoa abnormalities as previously described^59^. At least, 200 spermatozoa were microscopically examined for each sample.

### Hormonal assays

Hormonal assays were performed for *Brucella*-seropositive camel bulls (*n* = 11), and some randomly selected *Brucella*-seronegative camel bulls (*n* = 11). Serum testosterone levels were analyzed by enzyme immunoassay ELISA using the commercially available kits (DRG^®^Diagnostic, GmbH, A BioCheck Company, Marburg, Germany distributed by Clinilab, Cairo, Egypt)^60,61^. Serum cortisol levels were analyzed using the commercially available DRG Cortisol ELISA kits (DRG^®^ Diagnostic, GmbH, A BioCheck Company, Marburg, Germany distributed by Clinilab, Cairo, Egypt).

### Biochemical analysis

Serum levels of SOD and ascorbic acid were determined using the commercially available kits (Bio diagnostics, Cairo, Egypt). Serum levels of the APP; haptoglobin was determined using immunoturbidimetry commercially available kits (HP3222; Ben-Biochemical Enterprise S.r.l.-via Toselli, 4-20127 Milano Italy)^62–64^, and fibrinogen was determined using immunoturbidimetry commercially available kits (Salucea, Haansberg 19, 4874 NJ Etten Leur, The Netherlands, Cat. No. NS 590 001)^12,631]^.

### Histopathological preparation of the testicular tissues

Testicular tissues of *Brucella*-seropositive camel bulls (*n*= 4) were taken and fixed in 10% neutral buffered formalin for histopathological investigation. After 72 h of fixation, the tissue samples were gradually dehydrated by immersion in ascending concentrations of ethyl alcohol (70% I, 70% II, 70% III, 85%, 95%, 100% I, and 100% II), and cleared twice in methyl benzoate (1 h /each), and then infiltrated with paraffin for three hours. After being embedded in paraffin wax, the samples were sectioned (3 μm) for haematoxylin and eosin (H&E) staining^65^. Histopathological images were taken by using Leica EC3 digital camera.

### Statistical analysis

Raw data was entered and manipulated using Microsoft Excel spreadsheet, Microsoft Office 365 ProPlus. Data were expressed as mean ± SEM. Statistical analysis was conducted using the computer statistics Prism 6.0 package (GraphPad Software, Inc.). Statistical significance was determined by student’s t-test. Statistically significant differences values were set at *P* < 0.05.

Pearson correlations among all parameters were statistically analyzed in all the examined bulls, *Brucella*-seropositive and *Brucella*-seronegative bulls using the computer statistics Prism 6.0 package (GraphPad Software, Inc.) *P*-values less than 0.05 were considered statistically significant. *P < 0.05, **P < 0.01, and ***P < 0.001. Testicular histopathological findings were described qualitatively representing the descriptive histopathology of the affected tissues.

### ARRIVE guidelines

The present study was conducted according to the ARRIVE guidelines and all methods were conducted following relevant guidelines and regulations.

## Electronic supplementary material

Below is the link to the electronic supplementary material.

Supplementary Material 1

Supplementary Material 2

## Data Availability

The datasets analyzed and/or generated during this current study are available upon a reasonable request to the corresponding author.
