# Supplementary figures and images for: Poor semen quality is associated with impaired antioxidant response and acute phase proteins and is likely mediated by high cortisol levels in Brucella-seropositive dromedary camel bulls
Source: Sci Rep. 2024 Nov 13;14:27816. doi: 10.1038/s41598-024-74018-y (PMC11561072; doi:10.1038/s41598-024-74018-y)

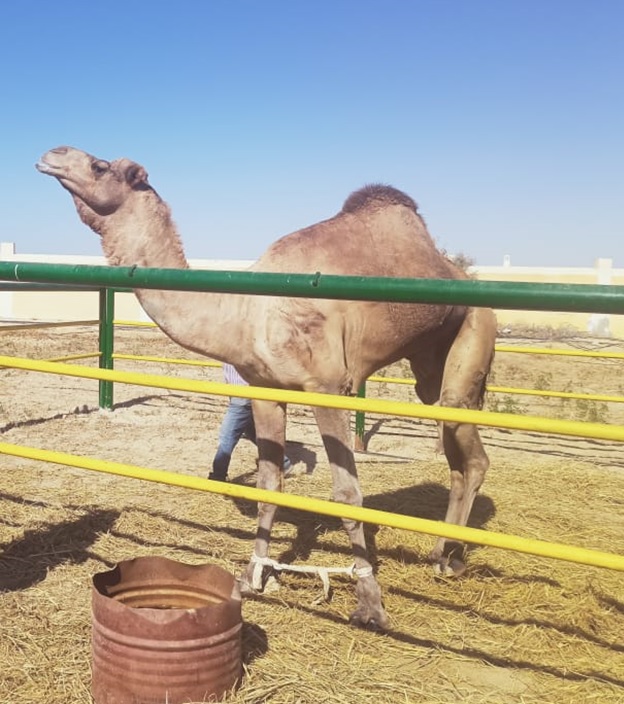

Supplement: Supplementary file 1 — Supplementary Material 1 [file 41598_2024_74018_MOESM1_ESM.jpg]
