# Supplementary material for: Poor semen quality is associated with impaired antioxidant response and acute phase proteins and is likely mediated by high cortisol levels in Brucella-seropositive dromedary camel bulls
Source: Sci Rep. 2024 Nov 13;14:27816. doi: 10.1038/s41598-024-74018-y (PMC11561072; doi:10.1038/s41598-024-74018-y)
Supplement: Supplementary file 2 — Supplementary Material 2 [file 41598_2024_74018_MOESM2_ESM.docx]

**Supplementary Figure S1.** A representative image for a Dromedary Arabian (one-humped) camel bull used in this study. The image shows the anatomical features of the Arabian camels with presence of characteristic one hump.
